# The Applied Data Analytics in Medicine Program: Lessons Learned From Four Years’ Experience With Personalizing Health Care in an Academic Teaching Hospital

**DOI:** 10.2196/29333

**Published:** 2022-01-28

**Authors:** Saskia Haitjema, Timothy R Prescott, Wouter W van Solinge

**Affiliations:** 1 Central Diagnostic Laboratory University Medical Center Utrecht Utrecht University Utrecht Netherlands; 2 Department of Digital Health University Medical Center Utrecht Utrecht University Utrecht Netherlands

**Keywords:** digital health, data-driven care, multidisciplinarity, lessons learned, eHealth, personalized medicine, data analytics, implementation, collaboration, hospital

## Abstract

The University Medical Center (UMC) Utrecht piloted a hospital-wide innovation data analytics program over the past 4 years. The goal was, based on available data and innovative data analytics methodologies, to answer clinical questions to improve patient care. In this viewpoint, we aimed to support and inspire others pursuing similar efforts by sharing the three principles of the program: the data analytics value chain (data, insight, action, value), the innovation funnel (structured innovation approach with phases and gates), and the multidisciplinary team (patients, clinicians, and data scientists). We also discussed our most important lessons learned: the importance of a clinical question, collaboration challenges between health care professionals and different types of data scientists, the win-win result of our collaboration with external partners, the prerequisite of available meaningful data, the (legal) complexity of implementation, organizational power, and the embedding of collaborative efforts in the health care system as a whole.

## Introduction

The University Medical Center (UMC) Utrecht is one of the largest academic teaching hospitals in The Netherlands. It has been using an electronic health record (EHR) system since 2007 and has ample experience in unlocking these data. The use of these routine health care data in data-driven care provides an opportunity to further personalize health care. Therefore, the UMC Utrecht executed a hospital-wide innovation program to explore if analysis of routine care data can be used to directly improve health care for patients. Personalizing health care using data analytics and specifically artificial intelligence (AI) is demanding for any health care organization. We would like to share the most important lessons we have learned over the last 4 years to inspire and support others.

## Program Set-up

The Applied Data Analytics in Medicine (ADAM) program started in 2017 as a proving ground with funding from the UMC Utrecht Board of Directors with the goal to try to improve health care for patients, by using innovative data analytics tools to answer clinical questions based on available routine care data. The program was headed by a core team of 4 people, who were ultimately responsible for the results. ADAM was based on three basic concepts: the value chain, the innovation funnel, and the multidisciplinary team

### The Data Analytics Value Chain

The value chain runs from data to insight to action to value. For example, when heart rhythm is captured (data), an absent heart rhythm can be found (insight), which warrants resuscitation (action) to save a life (value). Without action, value cannot be created.

### The Innovation Funnel

The innovation funnel consists of 7 phases with specific goals. In between the funnel phases, go and no-go moments can be found. The innovation funnel facilitated business-driven, controlled development for the project teams and prevented them from deviating from the clinical question. More importantly, the funnel provided the possibility to discontinue projects in an early stage of development. On an organizational level, the innovation funnel provided insight into the stage of development of the various data analytics projects.

### The Multidisciplinary Team

The multidisciplinary team always consisted of a patient, a health care professional, and a data scientist. The health care professional was the product owner of the data analytics project.

Data analytics proposals were prioritized based on relevance, feasibility, and scalability. In addition, educational value to contribute to a learning digital health care organization as a whole was a selection criterion. The multidisciplinary project team met weekly to discuss progress. Furthermore, cross-team meetings were organized to support interaction between teams. The project team organized project-specific expert sessions to discuss the project in a larger meeting with health care professionals or patients. To leverage lessons learned both within and outside the health care field, the program was established as a collaboration with different external business partners. As the UMC Utrecht only employed a few data scientists at the time, other parties (eg, external partners) provided data science expertise. The use of AI was optional and was seen as a means to an end. The program was extended twice, and total of 11 projects and 9 different external partners collaborated over 4 years’ time (Table 1). We organized 3 public seminars to spread our knowledge, and the infrastructure, activities, and interactions within the program provided the basis for 5 master’s degrees in fields other than health care.

**Table 1:** Projects of the Applied Data Analytics in Medicine (ADAM) program.

Specialty	Clinical goal
Cardiology	To support shared decision-making in cardiovascular risk management using a dashboard within the electronic health record system [1]
Rheumatology	To support the decision of whether or not to taper medication, based on risk of rheumatoid arthritis flares [2]
Psychiatry	To support a more personalized choice of antipsychotic medication
Neonatology	To support the decision to start antibiotics without a positive blood culture, as an early warning for neonatal sepsis
Microbiology	To support the decision to obtain a urine sample to lower unnecessary cultures
Gynecology	To support planning schedules in both the neonatal intensive care unit and the maternity ward by predicting capacity
Radiology	To obtain the infrastructure (hardware and software) to be able to apply artificial intelligence in radiology
Anesthesiology	To prioritize patients based on a visualized patient status overview
Intensive care medicine	To support ICU^a^ planning by predicting ICU capacity
Neonatology	To support the decision of whether or not to start a procedure on a neonate based on sleeping patterns

^a^ICU: intensive care unit.

## Lessons Learned

### Lesson 1. Clinical Practice as Both a Starting and End Point

Each data analytics project was based upon a relevant clinical question, provided by a health care professional [3]. We stimulated focus on this question by making the health care professional the product owner of the multidisciplinary project team. At the same time, this guaranteed the commitment of the health care professional. Moreover, the health care professionals received partial dispensation from clinical duties to participate in the program. In addition, the IT (information technology) department was involved from the start of the program, to facilitate expertise on data architecture and infrastructure that enabled both data extraction and model development, as well as interaction with the EHR system.

### Lesson 2. Health Care Professionals and Data Scientists Do Not Speak the Same Language

Health care professionals and data scientists each brought in specific expertise within their own frame of reference [4]. We therefore provided an objective project lead to further encourage collaboration. The project lead, for example, asked clarifying questions about the meaning of specific terms. We deliberately spent a considerable amount of time explaining medical processes and medical terminology to data scientists, for example, by drawing on the diagnostic or therapeutic process (where does the decision support system fit in this scheme? What decision will be ultimately supported?). A visit to the outpatient clinic or hospital ward proved to be of great value.

### Lesson 3. Data Professionals With Varying Expertise Are Needed

Data scientists in health care often choose a health care job consciously and are idealistic. One of the most important ways of keeping them excited was to provide them with diverse and challenging tasks: our data scientists worked on different projects at the same time, and we valued and encouraged the team spirit. We soon discovered that more roles were needed in addition to data scientists. Considerable effort was needed to collect and clean the data to preprocess it for data analysis (data stewards, data engineers). We also needed software developers, as well as UX (user experience) and UI (user interface) designers later on during development.

### Lesson 4. External Partners Are Partners, Not Just Suppliers

In our collaboration with external partners, we always aimed for a win-win situation. Partners pointed out the strategic benefits of working together with a hospital, indicating their willingness to invest in the relationship. All of our data scientists worked in the same project room. Because some partners provided company-specific expertise, this was a challenging way of collaborating. It was therefore crucial to discuss, for example, intellectual property before the project started (intellectual property was in principle allocated to the UMC Utrecht).

### Lesson 5. Available Data Need to Be Unlocked in a Meaningful Way

The collection of data in health care nowadays takes place in closed electronic health care systems that are not designed to enable reuse of data. The data need to be unlocked and, to prevent the “garbage in = garbage out” phenomenon, transformed into meaningful data in a workable format. In making these data analysis tables, domain knowledge, as well as knowledge of the medical process and technical knowledge of the EHR structure, are needed. Data stewards with combined knowledge were a valuable addition to the multidisciplinary teams. For example, we could build on the ample experience and available data from the Utrecht Patient Oriented Database [5]. In addition, sufficient data were needed for both epidemiologic determinants, as well as outcomes. In the project that aimed to personalize antipsychotic therapy, we, for example, couldn’t find a way to determine therapeutic success from the EHR, so the project was discontinued after the exploration phase. AI was not always needed for a successful project; for example, the successful model to predict flares in rheumatology was based on a joint modeling technique from classic epidemiology [2].

### Lesson 6. Implementation Is Versatile and Complex

If insights do not lead to action, value cannot be created and the data analytics project is essentially a waste of effort. Implementation of data analytics tools requires organizational changes that must not be underestimated. The multidisciplinary team, the expert sessions, and a UX researcher supported the changing health care processes at the UMC Utrecht. As the health care sector has fully embraced the concept of shared decision-making, the action that will be taken is the result of a shared decision. We therefore involved patients from the start of the clinical decision support tool, which supplemented the projects with important alternative perspectives. In addition, medical technology is always ahead of laws and regulations, for example, in the development of medical devices. Several partner departments in our hospital were of great value by pointing out the scope and leeway of legislation to support innovations. Next to ”is this possible?” and “is this allowed?” we always answered the ethical question “do we want this?” in our projects.

### Lesson 7. Organizational Power Is Essential

A large academic hospital is home to a lot of expertise, yet this often leads to a bureaucratic decision-making process. We had the advantage of being a program under the direct supervision of our Board of Directors, with a core team that had the authority to make decisions and the funding to carry them out. We were therefore prioritized by partner departments such as legal, privacy, and ethics. Without organizational power, it is difficult to run successful innovative projects.

### Lesson 8. You Cannot Do Anything Alone, You Have to Do It (Carefully) Together

Data analytics projects demand far-reaching collaboration. Health ministries could serve to bring inspiration and support to the current innovative health care climate. Academic medical centers should reach out and connect to smaller institutions because, for example, their academic population leads to underrepresentation of certain patient groups. Health care insurance companies should consider investing in projects that do not yield value directly to hospitals but are of value to the health insurance premium payers or society as a whole. The health care sector should find a way to collaborate with external partners that are for-profit, yet also possess a valuable network to valorize swiftly. As an example, the ADAM project IMAGR closed a contract to bring to the market a platform for radiology analysis using AI that connects to various existing radiology systems [6].

The experiences of our program led to the establishment of a new digital health department in the UMC Utrecht in early 2020. This department functions as a “hub” for digital health in the broadest sense, to further encourage and support the digital transition of the UMC Utrecht. The new department specifically appeals to the clinical departments: this is where medical processes take place and where data analytics is implemented in a responsible way. The positioning of the new department, close to the Board of Directors, provides the organizational power to make this happen.

Patients nowadays expect to be able to use personalized services always and everywhere. In 1959, the first article was published in which the author discussed computers supporting clinical decision-making [7]. Now, over 50 years later, it is about time for a flexible, digitally supported health care organization that facilitates personalized care at the right time and the right place, perhaps not with a personal computer on the doctor’s desk but with a mobile phone in hand.

## Abbreviations

ADAM: Applied Data Analytics in Medicine
AI: artificial intelligence
EHR: electronic health record
IT: information technology
UI: user interface
UMC: University Medical Center
UX: user experience
